# Still a place for aortic counterpulsation in cardiac surgery and patients with cardiogenic shock?

**DOI:** 10.1186/s13054-021-03673-8

**Published:** 2021-08-31

**Authors:** Matthias Heringlake, Astrid Ellen Berggreen, Hauke Paarmann

**Affiliations:** Department of Anesthesiology and Intensive Care Medicine, Heart and Diabetes Center, Mecklenburg-Western Pomerania, Karlsburg Hospital, Karlsburg, Germany

## Abstract

This article is one of ten reviews selected from the Annual Update in Intensive Care and Emergency Medicine 2021. Other selected articles can be found online at https://www.biomedcentral.com/collections/annualupdate2021. Further information about the Annual Update in Intensive Care and Emergency Medicine is available from https://link.springer.com/bookseries/8901.

## Introduction

Since its introduction into clinical practice in 1967 [1], the intra-aortic balloon pump (IABP) has played a prominent and steadily increasing role in cardiovascular medicine as the most frequently used mechanical circulatory support device. However, since the publication of a neutral Shock II trial on the effects of aortic counterpulsation in patients with myocardial infarction complicated by cardiogenic shock [2], use of this technology has decreased tremendously in many countries. It is of note that this decline has been observed not only in the field of cardiology—a finding that may easily be explained by guideline recommendations more or less prohibiting the use of an IABP in cardiogenic shock [3]—but also in cardiac surgery. In many European cardiac surgery centers, the IABP has been more or less completely substituted by other mechanical circulatory support modalities like the Impella^®^ or—more frequently—by veno-arterial extracorporeal life support (ECLS) systems. Unfortunately, the clinical results with both technologies are more than disappointing and show an unacceptably high mortality rate [4–6]. This finding is in clear contrast to several meta-analyses [7, 8] highlighting the beneficial effects on clinical outcomes of preemptive use of an IABP in cardiac surgery and an increasing number of publications showing beneficial hemodynamic and outcome effects of the IABP in cardiogenic shock [9–11].

The present chapter gives an overview of the effects of aortic counterpulsation in patients with cardiogenic shock and in patients with reduced myocardial function undergoing cardiac surgical procedures.

## Technological aspects and (patho-)physiological effects

The technological basis of aortic counterpulsation has been detailed recently [12]. Briefly, an IABP-system consists of a driving console and a helium-filled balloon that is usually inserted via the femoral route, and positioned into the descending aorta with the tip of the catheter just below the left subclavian artery. Triggered either by the electrocardiogram (EKG) or the arterial pressure curve derived from an integrated pressure line, the balloon is inflated during the diastolic part of the cardiac circle immediately after aortic valve closure and deflated just before the aortic valve opens again during ventricular systole, leading to an increase in diastolic pressure (and thereby coronary perfusion), and a reduction in left ventricular (LV) afterload [12]. Alternative insertion modalities may be used in which the balloon is directed in an antegrade fashion via the ascending aorta (typically in a patient with severe peripheral artery disease needing IABP-support for weaning from cardiopulmonary bypass [CPB]) or via the left axillary artery for prolonged support in patients with end-stage heart disease awaiting transplantation or implantation of a LV assist device (LVAD).

In patients with reduced LV ejection fraction (LVEF), intraaortic counterpulsation had a pronounced effect on cardiovascular dynamics as determined from a leftward shift of the pressure-volume curve associated with an increase in stroke volume and a reduction in LV end-diastolic pressure [12]. It is of note that the increase in stroke volume depends on the balloon volume used [13] and the compliance of the arterial system [14]. Consequently, increasing balloon size from the usual size of 30 or 40–50 ml leads to an increase in stroke volume and a more pronounced decrease in LV filling pressure [13]. In contrast, higher arterial compliance will render diastolic augmentation and afterload reduction during LV ejection less effective [14].

Unfortunately, since the diameter of the descending aorta is a natural limit, balloons with higher volumes are slightly longer than low volume balloons and may thus—even if the tip of the catheter is correctly positioned 1 cm below the orifice of the left subclavian artery—extend beyond the celiac trunk or even the renal arteries and thereby—at least if inflated—occlude these visceral arteries. Consequently, adequate sizing of the balloon is crucial to avoid decreased intestinal perfusion. To appropriately size the balloon, an equation based on age, height, sex, and the distance between the jugular notch and the symphysis has been suggested, by which the distance between the left subclavian artery and the celiac axis (LSA-CA) can be calculated and the optimal balloon size may be chosen [15]. Recently a specifically designed ‘short’ balloon has been developed that may overcome this problem [16]. Unfortunately, this balloon has not been tested in larger patient series.

## Intraaortic counterpulsation in cardiogenic shock

After introduction into clinical practice [1], observational trials in the pre-percutaneous coronary intervention (PCI) era revealed beneficial effects of intra-aortic counterpulsation on hemodynamics, metabolism, kidney function, and mortality in patients with cardiogenic shock [17, 18]. Based on these observations, the 2008 version of the European Society of Cardiology (ESC) guideline on the management of cardiogenic shock gave a class 1 level C recommendation to use the IABP in the management of this condition [19].

As noted earlier, this perspective has completely changed following the IABP-Shock II trial [2], and the current ESC-guideline on the management of acute heart failure now states that the IABP is not routinely recommended in cardiogenic shock due to myocardial infarction (class III, level B) [3]. Moreover, the guideline authors state that there are also sparse data to support the use of aortic counterpulsation in other clinical settings. Thus it is far from astonishing that the use of IABPs has decreased tremendously in cardiology practice [20, 21].

**Fig. 1:**
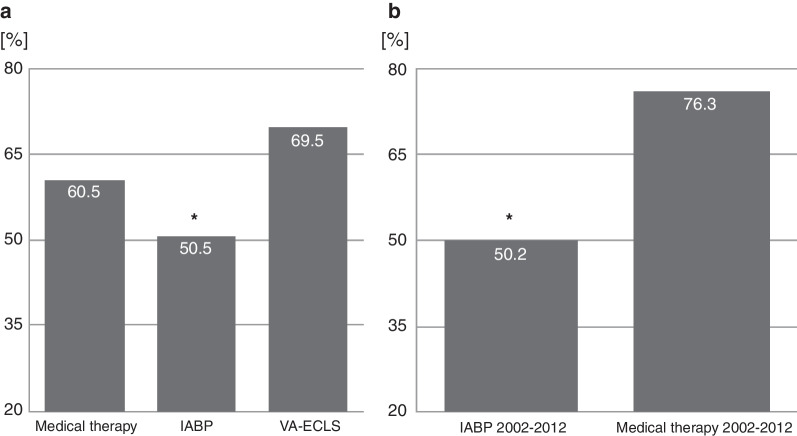
The effects of different treatment strategies on hospital mortality in patients with cardiogenic shock. **a** Hospital mortality from cardiogenic shock (based on ICD-10 code R57.0 as a main or secondary diagnosis) derived from the German Research Data Center of the Federal Bureau of Statistics (DESTATIS). Data are based on 333,459 patients treated medically, 36,805 patients treated with an intra-aortic balloon pump (IABP), and 9774 patients treated with veno-arterial extracorporeal life support (VA-ECLS) from Ref. [20]. **b** Hospital mortality from cardiogenic shock derived from the “Acute Coronary Syndrome Israeli Survey” (ACSIS) in 428 patients with cardiogenic shock treated with an IABP (*n* = 217) or medically (*n* = 211) from Ref. [21]. *Significant difference between medical and IABP treatment

Interestingly, a recent analysis of a German register for health outcomes showed that some centers continued to treat cardiogenic shock patients with an IABP and that these patients had a higher survival rate than patients managed conservatively or with other mechanical support systems (Fig. 1) [20]. Unfortunately, these data were not adjusted for disease severity and etiology of shock and may thus be subject to confounding. Interestingly, data from Israel, prospectively sampled in the Acute Coronary Syndrome Israeli Survey (ACSIS), point in the same direction and show that cardiogenic shock patients were frequently treated with an IABP from 2002 to 2012 (the year the IABP-Shock II data led to a downgrading of the IABP in the ESC guidelines) and had a significantly lower mortality than did conventionally treated patients (Fig. 1) [21]. Taking a detailed look at the IABP Shock II trial [2] and recent data analyzing the hemodynamic effects of the IABP in cardiogenic shock—with and without myocardial infarction—[9], these observations sound rather plausible.

Multiple criticisms of the study design and performance were raised after the publication of the IABP-Shock II trial [2]. However, to the best of our knowledge, the limitation of inadequate statistical power has not been discussed. Nevertheless, the power analysis of this trial was based on a mortality rate more than twice as high as that observed in the IABP-Shock trial [22], a sort of pilot trial for the IABP— Shock II study [2]. Consequently, at least based on the results of the *per protocol* analysis (showing a mortality rate of 36.5% in the IABP and 41.4% in the control group), the IABP-Shock II study would have shown a significant mortality benefit of the IABP if the trial had been powered according to the pilot trial that revealed a mortality of only 28.6% in the control group [22] instead of the 56% that was used to calculate the necessary sample-size for IABP-Shock II [2]. 

The recent literature on intraaortic counterpulsation reveals that there has been renewed interest in this technology and that the IABP seems far from outdated. Very recently, Malick and coworkers retrospectively analyzed the hemodynamic effects of intra-aortic counterpulsation in cardiogenic shock patients with acute myocardial infarction and acute decompensated heart failure and observed that the heart failure patients showed a significantly more pronounced increase in cardiac output in comparison with the myocardial infarction patients; the majority of patients with acute decompensated heart failure increased cardiac output, some even up to 3 l/min. Filling pressures decreased comparably in both patient groups [9]. The pathophysiological basis for this difference in efficacy remains speculative, but may be related to the vasodilatation often observed in patients with cardiogenic shock from acute myocardial infarction [23].

By contrast, several recent observational studies support the notion that aortic counterpulsation is beneficial not only in patients with acute decompensated heart failure and severely reduced myocardial function but also in cardiogenic shock and acute myocardial infarction. Gul and coworkers reported on a series of patients with cardiogenic shock (70% with acute coronary syndrome; 30% with other causes) with an overall mortality rate of 36.3% [10]. However, if an IABP was implanted within 1 h after admission, mortality was only 24% in comparison with 49% if the pump was inserted later. These findings are in line with another recent observational trial in 57 patients with reduced ejection fraction admitted with a systolic blood pressure < 100 mmHg. Patients treated early with an IABP had significantly lower 30-day mortality than patients who received the IABP later or were not treated with counterpulsation [24].

Den Uil et al. performed a small single center study comparing the effects of IABP-treatment (with a 50 ml balloon) compared to inotropes (enoximone or dobutamine) on mixed venous oxygen saturation (SvO2) in patients with decompensated heart failure and low cardiac output and showed that SvO2 normalized within 3 h in patients treated with an IABP but not in patients treated with inotropes. Ninety-day mortality in the inotropic group was twice as high as in the IABP group, but this failed to reach statistical significance due to the small sample size [11].

A propensity-matched comparison study analyzed the effects of IABP versus a micro-axial LVAD (Impella^®^) in patients with acute myocardial infarction and car-diogenic shock and observed that the mortality was almost significantly higher with the Impella^®^ than with the IABP (45% vs. 34%) and that bleeding complications were twice as high with the Impella^®^ [6].

The observational data presented so far show, that—despite appropriate and early use of an IABP—a certain number of patients cannot be adequately stabilized with this technology and may need to be resuscitated using extracorporeal veno-arterial perfusion [9, 10]. This, however, is associated with an increase in afterload of the failing left ventricle and may not only lead to an increased myocardial work and oxygen consumption but also sometimes to disastrous complications, such as intraventricular thrombosis. There is ongoing debate on the optimal mode to unload the left ventricle during ECLS. However, some recent data show that the concomitant use of an IABP during veno-arterial ECLS is an effective way to unload the left ventricle and has comparable efficacy to that of the Impella^®^ system [25].

Taken together, the observational data suggest that patients with acute decom-pensated heart failure and cardiogenic shock may benefit from aortic counterpulsation. Moreover, several trials contradict the neutral results of the IABP-Shock II trial [2] and show that the early use of an IABP may improve outcomes from cardiogenic shock complicating acute myocardial infarction. However, based on the observations of Malick and coworkers [9] this benefit may be restricted to patients presenting with increased systemic vascular resistance and supports the need to found the decision to start aortic counterpulsation on robust hemodynamic data. If an ‘upgrade’ to veno-arterial ECLS becomes inevitable, the IABP may still be used to ‘unload’ the left ventricle [25].

## Intraaortic counterpulsation in cardiac surgery

For many years, aortic counterpulsation was the modality of choice for mechanical support in cardiac surgery patients. Since the publication of the IABP-Shock II trial [2], the use of IABPs has also decreased in cardiac surgery, and many institutions now mostly rely on veno-arterial ECLS to support patients who cannot be weaned from CPB or only when using excessive doses of inotropes and vasopressors. There are no convincing prospective data available to support the use of ECLS in cardiac surgery patients. Moreover, meta-analyses suggest that the use of ECLS in cardiac surgery, even in experienced centers, is associated with an unacceptably high hospital mortality rate that is rarely below 60% [4].

By contrast, multiple meta-analyses support the notion that the preemptive, pre-operative implantation of an IABP in high-risk patients reduces mortality [7, 8]. Based on this, a German S3-guideline on the use of the IABP in cardiac surgery recommends that hemodynamically stable, high-risk cardiac surgery patients should be treated with intra-aortic counterpulsation, and that insertion should be performed preoperatively and before induction of anesthesia (grade of recommendation B, level of evidence 1b) [26].

There has been some criticism of these guidelines, because the studies included in the meta-analyses were small and monocenter, and several were performed by only one group of investigators. Additionally, two more recent trials [27, 28] failed to show a difference in the primary endpoint when comparing treated patients with a control group not supported by intra-aortic counterpulsation. However, in these studies, the methods clearly state that the IABP was switched off during CPB and therefore patients in the intervention group were devoid of an important effect of intra-aortic counterpulsation in cardiac surgery: the induction of pulsatility during CPB.

Several medium sized but elegantly performed studies have shown that induction of pulsatility by an IABP improves visceral and renal perfusion and thereby ameliorates the deleterious effects of non-pulsatile flow during CPB (overview in: [26]). Serraino and coworkers studied 501 patients in two groups—one supported by IABP during CPB and a control group with standard perfusion—and showed that IABP-pulsatile flow stabilized creatine clearance perioperatively and significantly reduced the incidence of grade 3 acute kidney injury from 20.4 to 7.8% [29]. Based on these findings, the German S3-guideline recommends that “upon preoperative insertion of an IABP this should be used to induce pulsatile blood flow during cardiopulmonary bypass” (grade of recommendation: A, level of evidence: 1b) [26].

In contrast to preoperative use, the intra- or postoperative use of an IABP— despite sometimes helpful to avoid an escalation to more invasive forms of mechanical support—has been associated with increased mortality [30]. Thus it is important to note that the German S3-guideline—based on the available literature—recommends a preemptive, prophylactic approach in a patient that typically has a normal or elevated systemic vascular tone. In line with the data of Malick et al. [9], this may help to avoid the insertion of an IABP when systemic vascular resistance is reduced (as is typically the case at the end of a long CPB run).

## Conclusion

Taken together, there seems to still be a place for intra-aortic counterpulsation in cardiogenic shock and cardiac surgery. Unfortunately, large scale trials supporting this technology are still missing. This may be explained by the fact that public funding organizations (and their reviewers) categorize the technology as outdated and useless (mostly based on the findings of the IABP-Shock II trial [2]); however—and especially in countries like Germany in which invasive technologies like ECLS are largely reimbursed [31]—clinical and industrial interests are now focusing on more invasive mechanical and life support technologies. Nonetheless, as recently proposed in an editorial, “the tide seems to be turning” and “there is some sun on the horizon regarding the use of the IABP” [32].

## Data Availability

Not applicable.
